# Are diet–prostate cancer associations mediated by the IGF axis? A cross-sectional analysis of diet, IGF-I and IGFBP-3 in healthy middle-aged men

**DOI:** 10.1038/sj.bjc.6600946

**Published:** 2003-05-27

**Authors:** D Gunnell, S E Oliver, T J Peters, J L Donovan, R Persad, M Maynard, D Gillatt, A Pearce, F C Hamdy, D E Neal, J M P Holly

**Affiliations:** 1 Department of Social Medicine, University of Bristol, Canynge Hall, Whiteladies Road, Bristol BS8 2PR, UK; 2 Department of Health Sciences, University of York, Seebohm Rowntree Building, Heslington, York YO10 5DD, UK; 3 Division of Primary Health Care, University of Bristol, Cotham House, Cotham Hill, Bristol BS6 6JL, UK; 4 Division of Surgery, University of Bristol, Bristol BS2 8HW, UK; 5 MRC Social and Public Health Sciences Unit, 3–5 Islington High St., London N1 9LQ, UK; 6 Academic Urology Unit, University of Sheffield, Sheffield S10 2JF, UK; 7 Oncology Centre, Addenbrooke's Hospital, Box 193, Hills Road, Cambridge CB2 2QQ, UK

**Keywords:** prostate cancer, IGF-I, IGFBP-3, diet, epidemiology

## Abstract

We examined the association of diet with insulin-like growth factors (IGF) in 344 disease-free men. Raised levels of IGF-I and/or its molar ratio with IGFBP-3 were associated with higher intakes of milk, dairy products, calcium, carbohydrate and polyunsaturated fat; lower levels with high vegetable consumption, particularly tomatoes. These patterns support the possibility that IGFs may mediate some diet–cancer associations.

High circulating levels of insulin-like growth factor-I (IGF-I) are associated with an increased risk of developing prostate (Chan *et al*, 1998; Harman *et al*, 2000; Stattin *et al*, 2000; Chokkalingam *et al*, 2001) and other cancers (Holly *et al*, 1999). In serum and body fluids, IGF-I's activity is regulated by a complex system of six binding proteins and an acid-labile subunit. Most (90%) circulating IGF-I is bound to IGF binding protein-3 (IGFBP-3) and associations of IGFs with prostate cancer are generally strongest with the molar ratio (IGF-I/IGFBP-3) or in statistical models controlling for IGFBP-3 (Chan *et al*, 1998; Harman *et al*, 2000; Stattin *et al*, 2000; Chokkalingam *et al*, 2001). Raised levels of bioavailable IGF-I may, therefore, increase cancer risk, and raised IGFBP-3, by reducing IGF-I's bioavailability, may reduce risk.

IGF-I plays a role in energy and protein metabolism as well as modulating cell turnover and apoptosis (Thissen *et al*, 1994; Holly *et al*, 1999). Energy restriction leads to reduced production of IGF-I (Thissen *et al*, 1994), and animal experiments suggest that this pathway mediates the cancer-protective role of diet restriction (Dunn *et al*, 1997). Other dietary influences on IGFs may underlie some of the diet–prostate cancer associations observed. While no specific food or nutrient is an established risk factor for prostate cancer, dietary aspects most consistently related to its risk are red meat, animal fat, calcium and dairy product consumption and lower consumption of vegetables (Kolonel, 1996; World Cancer Research Fund, 1997; Department of Health, 1998). Diets rich in tomatoes, a major source of the carotenoid lycopene, are associated with reduced risk (Giovannucci, 1999).

Several, generally small, cross-sectional studies have examined the association of diet with the IGF axis (Darling-Raedeke *et al*, 1998; Kaklamani *et al*, 1999; Allen *et al*, 2000; Signorello *et al*, 2000; Mucci *et al*, 2001; Holmes *et al*, 2002; Giovannucci *et al*, 2003). The largest investigation (*n*=1037) (Holmes *et al*, 2002) reported that higher levels of energy, protein and milk intake were associated with raised IGF-I and high fat intake with low IGFBP-3, broadly consistent with previous, smaller, studies (Kaklamani *et al*, 1999; Ma *et al*, 2001). Other studies have reported reduced levels of IGF-I with tomato consumption (Mucci *et al*, 2001) and vegan diets (Allen *et al*, 2000). There has been only one (Giovannucci *et al*, 2003) large-scale investigation of the association of diet with IGF in community-based men.

## MATERIALS AND METHODS

Within a case–control study nested in a population-based investigation into the early detection and management of prostate cancer (ProtecT; Donovan *et al*, 2002), stored blood samples from 368 disease-free men (controls) were assayed for IGF-I and IGFBP-3. Controls were matched to cases on age, general practice and date of recruitment. Included in this analysis are the 344 (95%) of these disease-free men who completed a 114-item validated food-frequency questionnaire (FFQ) (Bingham *et al*, 1997). Over two-thirds (*n*=242) of the men also provided information on occupation, smoking and physical activity and had height and weight measured. Ethical approval was obtained from the relevant multicentre and local research ethics committees.

Based on FFQ responses, and using standard food tables (The Royal Society of Chemistry and MAFF, 1991) and portion size data for men of this age (Ministry of Agriculture and Food, 1993), we estimated weekly consumption of: energy, carbohydrate, protein, total fat, saturated and polyunsaturated fat, calcium, red meat, dairy products, vegetables, milk, tomatoes and foods containing tomatoes (baked beans, tomato ketchup and tomato juice). These were selected on the basis of research findings and reviews examining associations of diet with prostate cancer (World Cancer Research Fund, 1997; Giovannucci, 1999; Chan and Giovannucci, 2001) and the IGF-axis (Ma *et al*, 2001; Holmes *et al*, 2002). Given difficulties in measuring lycopene from FFQs (Kristal and Cohen, 2000), we used the frequency of reported consumption of tomatoes and products with high tomato content.

### Laboratory methods

Non-fasted blood specimens, taken using standard techniques, were spun and frozen to −80°C within 18 h. For the IGF-I assays, an ELISA kit was used (Diagnostic Systems Laboratories, TX, USA). Assays for serum IGFBP-3 used a previously validated ‘in-house’ double antibody radioimmunoassay (Cheetham *et al*, 1998). The average coefficients of variation for *intra*-assay variability for IGF-I and IGFBP-3 were 3 and 3.6%, and for *inter*-assay variation were 15 and 14%. To measure (crudely) bioavailable IGF-I, we multiplied the molar ratio of IGF-I/IGFBP-3 by 5.33 (molecular weights 40 000 and 7500 Da, respectively).

### Statistical analysis

Using Stata (Stata Corporation, 2001) we calculated age-, centre- and energy-adjusted levels of IGF-I, IGFBP-3 and the molar ratio in quartiles of the distribution of each dietary factor. Intakes of individual food groups were considered in three *a priori* categories. Adjustment for overall energy intake means that dietary measures relate to dietary composition rather than absolute intake (Willett, 1998).

Least-squares linear regression models investigated change in growth factor levels for a one standard deviation increase in each dietary factor. Log or square-root transformations were used for the latter due to positive skewness, and sampling weights adjusted for the dependence on the age distribution of cases. Tests for trend were based on the continuous variable (for nutrients and food groups) or three-level category (for tomato products and milk).

We assessed possible confounding by exercise, smoking, body mass index (BMI) and socioeconomic position in the 242 men with complete data.

## RESULTS

Mean age was 62.2 years (range 50–70) and most men (90%) were nonsmokers and came from nonmanual social classes (64%). Mean (s.d.) blood levels of IGF-I, IGFBP-3 and the molar ratio (IGF-I/IGFBP-3) were 126.6 ng ml^−1^ (36.9), 3393.6 ng ml^−1^ (1049.8) and 0.21 (0.08), respectively. Median daily intakes were as follows: energy: 10.3 MJ; carbohydrate: 314.8 g; protein: 89.5 g; fat: 77.6 g; red meat: 46.9 g; dairy products 344.9 g; calcium 1126.3 g; vegetables 271.0 g.

Raised IGF-I levels were seen in men consuming higher levels of polyunsaturated fat (*P*_trend_=0.017) and calcium (*P*_trend_=0.035) (Table 1

**Table 1:** Age-, centre- and energy-adjusted levels of IGF-I, IGFBP-3 and IGF-I/IGFBP-3 molar ratio in relation to quartiles of increasing intake of dietary variables (*n*=344)

	**Quartile**					
	**1 (low intake)**	**2**	**3**	**4 (high intake)**	**Change in growth factor (95% CI) per s.d. increase in each dietary variable**	***P*-value (linear trend)^b^**
IGF-I (ng ml^−1^)						
Energy intake (MJ)	119.9	133.8	129.3	125.8	1.2 (−3.6 to 5.9)	0.63
Carbohydrates (g)	109.2	128.6	129.6	141.5	10.0 (−1.2 to 21.2)	0.08
Protein (g)	115.7	137.4	131.5	124.9	2.6 (−6.5 to 11.8)	0.57
Fat (g)	121.1	125.4	125.3	137.2	5.0 (−4.6 to 14.6)	0.30
Polyunsaturated fat (g)	114.4	122.6	136.2	136.1	9.1 (1.6 to 16.5)	0.017
Saturated fat (g)	125.3	121.4	128.5	133.9	1.8 (−6.5 to 10.2)	0.67
Red meat (g)	125.2	134.2	135.3	117.8	−2.7 (−8.3 to 2.9)	0.35
Dairy products (g)	117.8	130.7	125.6	134.1	4.4 (−0.8 to 9.7)	0.09
Calcium (mg)	120.5	119.1	137.2	131.8	6.5 (0.5 to 12.5)	0.035
Vegetables (g)	134.1	125.9	122.8	126.5	−2.1 (−8.3 to 4.1)	0.50
						
IGFBP-3 (ng ml^−1^)						
Energy intake (MJ)	3281.6	3479.5	3494.2	3328.1	15.4 (−101.6 to 132.4)	0.80
Carbohydrates (g)	3017.2	3423.7	3541.0	3598.2	184.5 (−131.6 to 500.7)	0.25
Protein (g)	3120.7	3408.3	3651.0	3395.1	150.0 (−107.2 to 407.2)	0.25
Fat (g)	3564.5	3387.7	3299.6	3335.9	−85.8 (−329.8 to 158.3)	0.49
Polyunsaturated fat (g)	3272.0	3288.7	3357.0	3667.2	228.1 (−1.2 to 457.4)	0.05
Saturated fat (g)	3465.4	3514.0	3444.0	3162.4	−170.4 (−374.7 to 33.9)	0.10
Red meat (g)	3469.4	3389.2	3521.4	3196.7	−78.0 (−196.5 to 40.4)	0.20
Dairy products (g)	3348.8	3450.2	3385.1	3396.2	3.9 (−139.4 to 147.3)	0.96
Calcium (mg)	3345.5	3326.7	3540.2	3368.1	81.2 (−95.6 to 258.1)	0.37
Vegetables (g)	3164.8	3637.4	3401.5	3374.8	93.8 (−55.7 to 243.4)	0.22
						
IGF-I : IGFBP-3 molar ratio						
Energy intake (MJ)	0.206	0.221	0.210	0.213	−0.001 (−0.012 to 0.010)	0.85
Carbohydrates (g)	0.202	0.214	0.209	0.226	−0.009 (−0.034 to 0.016)	0.48
Protein (g)	0.204	0.231	0.204	0.213	−0.002 (−0.021 to 0.018)	0.88
Fat (g)	0.193	0.208	0.214	0.235	0.011 (−0.007 to 0.029)	0.23
Polyunsaturated fat (g)	0.200	0.212	0.231	0.208	−0.002 (−0.017 to 0.013)	0.84
Saturated fat (g)	0.208	0.196	0.210	0.238	0.012 (−0.006 to 0.030)	0.19
Red meat (g)	0.204	0.224	0.221	0.209	0.001 (−0.008 to 0.011)	0.82
Dairy products (g)	0.205	0.209	0.212	0.225	0.007 (−0.003 to 0.018)	0.18
Calcium (mg)	0.207	0.204	0.218	0.222	0.006 (−0.008 to 0.019)	0.39
Vegetables (g)	0.236	0.206	0.201	0.209	−0.011 (−0.022 to −0.000)	0.045

a All values are controlled for age, study centre and energy intake, except that for energy intake, which is controlled for age and study centre only. All models are weighted by the inverse of the sampling probability in relation to age.

b Tests for trend based on continuous measure of diet.

). There were weaker positive associations with carbohydrate and dairy products. IGFBP-3 levels were weakly positively associated with polyunsaturated fats (*P*_trend_=0.05) and inversely associated with saturated fats (*P*_trend_=0.10). The molar ratio was inversely related to vegetable intake (*P*_trend_=0.045).

Controlling for BMI, social class, smoking and exercise attenuated the associations of IGF-I and IGFBP-3 with carbohydrates, polyunsaturated fats and, to a lesser extent, vegetables (not shown). Associations of dairy products and calcium with IGF-I and saturated fat with IGFBP-3 were not confounded.

IGF-I tended to be lower and IGFBP-3 higher in those who ate tomatoes or tomato-containing products more frequently, although evidence for a trend was only clear for IGF-I/IGFBP-3 molar ratio (Table 2

**Table 2:** Age-, centre- and energy-adjusted mean levels of IGF-I, IGFBP-3 and IGF-I/IGFBP-3 molar ratio in relation to increasing levels of intake of tomatoes, tomato-rich products and milk

	**Weekly consumption**			
**IGF-I (ng ml^−1^) (no. with data)**	**<Once per week**	**1–4 times per week**	**5+ times per week**	***P*-trend**
Tomatoes (*n*=342)	139.2 (*n*=45)	125.3 (*n*=232)	126.3 (*n*=65)	0.19
Baked beans (*n*=341)	129.3 (*n*=139)	126.8 (*n*=196)	119.6 (*n*=6)	0.55
Tomato ketchup (*n*=343)	130.3 (*n*=233)	121.6 (*n*=100)	120.4 (*n*=10)	0.10
Tomato juice (*n*=343)	127.9 (*n*=324)	122.9 (*n*=14)	87.0 (*n*=5)	0.14
				
	**Daily consumption**			
	**<½ pint**	**½**−**34** **pint**	**1+ pints**	
Milk (*n*=342)	117.8 (*n*=95)	126.2 (*n*=173)	140.7 (*n*=74)	0.004
	**Weekly consumption**			
**IGFBP-3 (ng ml^−1^)**	**<Once per week**	**1–4 times per week**	**5+ times per week**	***P*-trend**
Tomatoes (*n*=342)	3648.8	3322.2	3504.9	0.70
Baked beans (*n*=341)	3377.9	3424.4	3743.0	0.63
Tomato ketchup (*n*=343)	3275.7	3732.1	2968.9	0.09
Tomato juice (*n*=343)	3383.1	3958.7	2715.9	0.47
				
	**Daily consumption**			
	**<½ pint**	**½**−**34** **pint**	**1+ pints**	
Milk (*n*=342)	3343.9	3394.6	3465.7	0.62
	**Weekly consumption**			
**IGF-I/BP-3 molar ratio**	**<Once per week**	**1–4 times per week**	**5+ times per week**	***P*-trend**
Tomatoes (*n*=342)	0.221	0.214	0.204	0.28
Baked beans (*n*=341)	0.214	0.212	0.177	0.64
Tomato ketchup (*n*=343)	0.224	0.187	0.223	0.005
Tomato juice (*n*=343)	0.214	0.178	0.177	0.004
				
	**Daily consumption**			
	**<½ pint**	**½**−**34** **pint**	**1+ pints**	
Milk (*n*=342)	0.206	0.209	0.230	0.17

a All values are controlled for age, study centre and energy intake and are weighted by the inverse of the sampling probability in relation to age.

). Men consuming higher levels of milk had raised levels of IGF-I (*P*_trend_=0.004). There was no association between milk intake and IGFBP-3 and the molar ratio was highest in those men drinking at least one pint of milk per day. These associations were little changed after adjustment for BMI, social class, smoking and exercise (not shown). Associations of IGF-I with milk were not confounded by calcium intake, whereas associations with calcium intake were attenuated in models controlling for milk intake.

Excluding men (*n*=95) reporting low levels of energy intake in relation to their estimated basal metabolic rate (ratio of energy intake/basal metabolic rate <1.2 (Joint FAO/WHO/UN Expert Consultation, 1985)) did not change the associations with milk intake, tomato-rich products or vegetable intake.

## DISCUSSION

In a group of healthy, community-sampled, middle-aged men, we found associations of the IGF-axis with several aspects of diet linked previously to prostate cancer. Positive relations were seen with dairy products, milk and calcium intake, all of which were associated with raised IGF-I levels. High intakes of vegetables and tomatoes or tomato-containing products were associated with lower levels of IGF-I or its molar ratio. In contrast to some other studies (Kaklamani *et al*, 1999; Holmes *et al*, 2002), we found only weak associations with saturated fat and no evidence of an association with red meat.

Associations were not confounded by socioeconomic position or lifestyle. While we have examined associations with a range of dietary variables and three different measures of the IGF-axis, thereby increasing the possibility of chance results, our findings are consistent with previous studies. In a cross-sectional study, it is not possible to determine whether dietary associations arise as the result of long-term intake of particular foods/nutrients or reflect patterns of intake around the time of blood sampling.

Association of IGF-I levels with dairy products, milk and calcium are consistent with some (Heaney *et al*, 1999; Ma *et al*, 2001; Holmes *et al*, 2002) but not all (Mucci *et al*, 2001) previous analyses. The strongest evidence of a causal association between higher levels of milk consumption and IGF comes from a randomised trial of dietary milk supplementation, reporting a rise in IGF-I in those supplemented but not the controls (Heaney *et al*, 1999). While some research suggests that neonates absorb IGF-I from breast milk (Diaz-Gomez *et al*, 1997), there is no strong evidence that bovine IGF-I in cows milk could be similarly absorbed from the gut (Holmes *et al*, 2002). Dietary intake of animal protein (essential amino acids) is known to stimulate IGF-I production (Thissen *et al*, 1994), but we found no evidence of associations with animal protein intake, nor that controlling for animal protein intake attenuated associations with milk (not shown). This contrasts with the findings of Giovannucci *et al* (2003) and Holmes *et al* (2002). In Giovannucci *et al*'s analysis, associations with vegetable protein were, however, of similar magnitude to those for animal protein.

Associations of calcium, milk and dairy products with IGF-I suggest a possible pathway linking dietary intake of these factors with prostate cancer (Chan and Giovannucci, 2001). The relation of these dietary aspects with prostate cancer risk are, however, in the opposite direction to their association with colorectal cancer (Ma *et al*, 2001; Wu *et al*, 2002), another neoplasm associated with raised IGF-I levels (Ma *et al*, 1999). The IGF–cancer associations seen for a range of different cancer sites (Holly *et al*, 1999; Yu and Rohan, 2000) may not therefore be explained in terms of common dietary influences on the growth factor axis. Nevertheless, our finding that vegetable intake was weakly related to lower molar ratios is consistent with the observation that vegetable-rich diets appear to protect against colorectal, breast and prostate cancer (World Cancer Research Fund, 1997), although associations of vegetable intake with IGF-I or IGFBP-3 have not been found in other studies (Kaklamani *et al*, 1999; Holmes *et al*, 2002).

The weak associations of IGFs with tomatoes and tomato-containing products support those reported for 112 Greek men (Mucci *et al*, 2001), where a strong inverse association was found between cooked tomato consumption and IGF-I. Likewise, in the Nurses Study intake of lycopene was positively associated with circulating levels of IGFBP-3 (but not IGF-I). These findings hint at the possible importance of the IGF axis in mediating the protective effect of higher levels of tomato or lycopene intake on prostate cancer reported in several investigations (Giovannucci, 1999). A possible biological mechanism lies in the reported inhibitory effects of lycopene on IGF-I receptor signalling and cell cycle progression (Karas *et al*, 2000), but a small trial of lycopene supplementation found no difference in IGF-I levels in supplemented *vs* control subjects (Kucuk *et al*, 2001).

International comparisons of cancer incidence and changes in incidence in migrants moving between different continents, indicates large dietary influences on epithelial cancer incidence (World Cancer Research Fund, 1997). Our study adds to evidence that aspects of diet previously linked to prostate cancer may influence cancer risk through the IGF-axis. Trials of dietary interventions aimed at reducing bioavailable IGF-I are now required. Identification of relevant aspects of diet could then lead to trials of dietary interventions against cancer incorporating measurements of IGF-I.

## References

[bib1] Allen NE, Appleby PN, Davey GK, Key TJ (2000) Hormones and diet: low insulin-like growth factor-I but normal bioavailable androgens in vegan men. Br J Cancer 83: 95–97 10883675 10.1054/bjoc.2000.1152 PMC2374537

[bib2] Bingham SA, Gill C, Welch A, Cassidy A, Runswick SA, Oakes S, Lubin R, Thurnham DI, Key TJA, Roe L, Khaw KT, Day NE (1997) Validation of dietary assessment methods in the UK arm of EPIC using weighed records, and 24-hour urinary nitrogen and potassium and serum vitamin C and carotenoids as biomarkers. Int J Epidemiol 26: S137–S151 9126542 10.1093/ije/26.suppl_1.s137

[bib3] Chan JM, Giovannucci EL (2001) Dairy products, calcium, and vitamin D and risk of prostate cancer. Epidemiol Rev 23: 87–92 11588859 10.1093/oxfordjournals.epirev.a000800

[bib4] Chan JM, Stampfer MJ, Giovannucci E, Gann PH, Ma J, Wilkinson P, Hennekens CH, Pollak M (1998) Plasma insulin-like growth factor-I and prostate cancer risk: a prospective study. Science 279: 563–566 9438850 10.1126/science.279.5350.563

[bib5] Cheetham TD, Holly JM, Baxter RC, Meadows K, Jones J, Taylor AM, Dunger DB (1998) The effects of recombinant human IGF-I administration on concentrations of acid labile subunit, IGF binding protein-3, IGF-I, IGF-II and proteolysis of IGF binding protein-3 in adolescents with insulin-dependent diabetes mellitus. J Endocrinol 157: 81–87 9614361 10.1677/joe.0.1570081

[bib6] Chokkalingam AP, Pollak M, Fillmore CM, Gao YT, Stanczyk FZ, Deng J, Sesterhenn IA, Mostofi FK, Fears TR, Madigan MP, Ziegler RG, Fraumeni Jr JF, Hsing AW (2001) Insulin-like growth factors and prostate cancer: a population-based case–control study in China. Cancer Epidemiol Biomarkers Prev 10: 421–427 11352850

[bib7] Darling-Raedeke M, Thornton Jr WH, MacDonald RS (1998) Growth hormone and IGF-I plasma concentrations and macronutrient intake measured in a free-living elderly population during a one-year period. J Am Coll Nutr 17: 392–397 9710852 10.1080/07315724.1998.10718782

[bib8] Department of Health (1998) Report on Health and Social Subjects Nutritional Aspects of the Development of Cancer. Norwich: Her Majesty's Stationery Office

[bib9] Diaz-Gomez NM, Domenech E, Barroso F (1997) Breast-feeding and growth factors in preterm newborn infants. J Pediatr Gastroenterol Nutr 24: 322–327 9138180 10.1097/00005176-199703000-00016

[bib10] Donovan J, Mills N, Smith M, Brindle L, Jacoby A, Peters T, Frankel S, Neal D, Hamdy F (2002) Quality improvement report: improving design and conduct of randomised trials by embedding them in qualitative research: ProtecT (prostate testing for cancer and treatment) study. Commentary: presenting unbiased information to patients can be difficult. BMJ 325: 766–770 12364308 10.1136/bmj.325.7367.766 PMC1124277

[bib11] Dunn SE, Kari FW, French J, Leininger JR, Travlos G, Wilson R, Barrett JC (1997) Dietary restriction reduces insulin-like growth factor I levels, which modulates apoptosis, cell proliferation, and tumor progression in p53-deficient mice. Cancer Res 57: 4667–4672 9354418

[bib12] Giovannucci E (1999) Tomatoes: tomato-based products, lycopene and cancer: review of the epidemiologic literature. J Nat Cancer Inst 91: 317–331 10050865 10.1093/jnci/91.4.317

[bib13] Giovannucci E, Pollak M, Liu Y, Platz EA, Majeed N, Rimm EB, Willett WC (2003) Nutritional predictors of insulin-like growth factor I and their relationships to cancer in men. Cancer Epidemiol Biomarkers Prev 12: 84–89 12582016

[bib14] Harman SM, Metter EJ, Blackman MR, Landis PK, Carter HB (2000) Serum levels of insulin-like growth factor I (IGF-I), IGF-II, IGF- binding protein-3, and prostate-specific antigen as predictors of clinical prostate cancer. J Clin Endocrinol Metab 85: 4258–4265 11095464 10.1210/jcem.85.11.6990

[bib15] Heaney RP, McCarron DA, Dawson-Hughes B, Oparil S, Berga SL, Stern JS, Barr SI, Rosen CJ (1999) Dietary changes favorably affect bone remodeling in older adults. J Am Diet Assoc 99: 1228–1233 10524386 10.1016/S0002-8223(99)00302-8

[bib16] Holly JMP, Gunnell DJ, Davey Smith G (1999) Growth hormone, IGF-I and cancer. Less intervention to avoid cancer? More intervention to prevent cancer. J Endocrinol 162: 321–330 10467223 10.1677/joe.0.1620321

[bib17] Holmes MD, Pollak MN, Willett WC, Hankinson SE (2002) Dietary correlates of plasma insulin-like growth factor I and insulin-like growth factor binding protein 3 concentrations. Cancer Epidemiol Biomarkers Prev 11: 852–861 12223429

[bib18] Joint FAO/WHO/UNU Expert Consultation (1985) Energy and Protein Requirements. Geneva: World Health Organisation 3937340

[bib19] Kaklamani VG, Linos A, Kaklamani E, Markaki I, Koumantaki Y, Mantzoros CS (1999) Dietary fat and carbohydrates are independently associated with circulating insulin-like growth factor 1 and insulin-like growth factor- binding protein 3 concentrations in healthy adults. J Clin Oncol 17: 3291–3298 10506632 10.1200/JCO.1999.17.10.3291

[bib20] Karas M, Amir H, Fishman D, Danilenko M, Segal S, Nahum A, Koifmann A, Giat Y, Levy J, Sharoni Y (2000) Lycopene interferes with cell cycle progression and insulin-like growth factor I signaling in mammary cancer cells. Nutr Cancer 36: 101–111 10798222 10.1207/S15327914NC3601_14

[bib21] Kolonel LN (1996) Nutrition and prostate cancer. Cancer Causes Control 7: 83–94 8850437 10.1007/BF00115640

[bib22] Kristal AR, Cohen H (2000) Invited commentary: tomatoes, lycopene, and prostate cancer. How strong is the evidence? Am J Epidemiol 151: 124–127 10645814 10.1093/oxfordjournals.aje.a010177

[bib23] Kucuk O, Sarkar FH, Sakr W, Djuric Z, Pollak MN, Khachik F, Li Y-W, Banerjee M, Grignon D, Bertram JS, Crissman JD, Pontes EJ, Wood DPJ (2001) Phase II randomized clinical trial of lycopene supplementation before radical prostatectomy. Cancer Epidemiol Biomarkers Prev 10: 861–868 11489752

[bib24] Ma J, Giovannucci E, Pollak M, Chan JM, Gaziano JM, Willett W, Stampfer MJ (2001) Milk intake, circulating levels of insulin-like growth factor-i, and risk of colorectal cancer in men. J Nat Cancer Inst 93: 1330–1336 11535708 10.1093/jnci/93.17.1330

[bib25] Ma J, Pollak MN, Giovannucci E, Chan JM, Tao Y, Hennekens CH, Stampfer MJ (1999) Prospective study of colorectal cancer risk in men and plasma levels of insulin-like growth factor (IGF)-1 and IGF-binding protein-3. J Natl Cancer Inst 91: 620–625 10203281 10.1093/jnci/91.7.620

[bib26] Ministry of Agriculture and Food (1993) Food Portion Sizes. Ref Type: Pamphlet. London: HMSO

[bib27] Mucci LA, Tamimi R, Lagiou P, Trichopoulou A, Benetou V, Spanos E, Trichopoulos D (2001) Are dietary influences on the risk of prostate cancer mediated through the insulin-like growth factor system. BJU Int 87: 814–820 11412218 10.1046/j.1464-410x.2001.02191.x

[bib28] Signorello LB, Kuper H, Lagiou P, Wuu J, Mucci LA, Trichopoulos D, Adami HO (2000) Lifestyle factors and insulin-like growth factor 1 levels among elderly men. Eur J Cancer Prev 9: 173–178 10954256

[bib29] Stata Corporation (2001) Intercooled Stata 7.0 for Windows. TX, USA: Stata Corporation

[bib30] Stattin P, Bylund A, Rinaldi S, Biessy C, Dechaud H, Stenman UH, Egevad L, Riboli E, Hallmans G, Kaaks R (2000) Plasma insulin-like growth factor-I, insulin-like growth factor-binding proteins, and prostate cancer risk: a prospective study. J Natl Cancer Inst 92: 1910–1917 11106682 10.1093/jnci/92.23.1910

[bib31] The Royal Society of Chemistry and MAFF (1991) McCance and Widdowson's The Composition of Foods. London: HMSO

[bib32] Thissen JP, Ketelslegers JM, Underwood LE (1994) Nutritional regulation of the insulin-like growth factors. Endocrine Rev 15: 80–101 8156941 10.1210/edrv-15-1-80

[bib33] Willett WC (1998) Nutritional Epidemiology. New York: Oxford University Press

[bib34] World Cancer Research Fund (1997) Food, Nutrition and the Prevention of Cancer: a Global Perspective. Washington, DC: American Institute for Cancer Research 10.1016/s0899-9007(99)00021-0 10378216

[bib35] Wu K, Willett WC, Fuchs CS, Colditz GA, Giovannucci EL (2002) Calcium intake and risk of colon cancer in women and men. J Natl Cancer Inst 94: 437–446 11904316 10.1093/jnci/94.6.437

[bib36] Yu H, Rohan T (2000) Role of the insulin-like growth factor family in cancer development and progression. J Natl Cancer Inst 92: 1472–1489 10995803 10.1093/jnci/92.18.1472

